# Two distinct pathways of pregranulosa cell differentiation support follicle formation in the mouse ovary

**DOI:** 10.1073/pnas.2005570117

**Published:** 2020-08-05

**Authors:** Wanbao Niu, Allan C. Spradling

**Affiliations:** ^a^Howard Hughes Medical Institute Research Laboratories, Carnegie Institution for Science, Baltimore, MD 21218;; ^b^Department of Embryology, Carnegie Institution for Science, Baltimore, MD 21218

**Keywords:** mouse, ovary, follicle, pregranulosa, scRNAseq

## Abstract

We sequenced more than 52,500 single cells from embryonic day 11.5 (E11.5) postembryonic day 5 (P5) gonads and performed lineage tracing to analyze primordial follicles and wave 1 medullar follicles during mouse fetal and perinatal oogenesis. Germ cells clustered into six meiotic substages, as well as dying/nurse cells. Wnt-expressing bipotential precursors already present at E11.5 are followed at each developmental stage by two groups of ovarian pregranulosa (PG) cells. One PG group, bipotential pregranulosa (BPG) cells, derives directly from bipotential precursors, expresses Foxl2 early, and associates with cysts throughout the ovary by E12.5. A second PG group, epithelial pregranulosa (EPG) cells, arises in the ovarian surface epithelium, ingresses cortically by E12.5 or earlier, expresses Lgr5, but delays robust Foxl2 expression until after birth. By E19.5, EPG cells predominate in the cortex and differentiate into granulosa cells of quiescent primordial follicles. In contrast, medullar BPG cells differentiate along a distinct pathway to become wave 1 granulosa cells. Reflecting their separate somatic cellular lineages, second wave follicles were ablated by diptheria toxin treatment of Lgr5-DTR-EGFP mice at E16.5 while first wave follicles developed normally and supported fertility. These studies provide insights into ovarian somatic cells and a resource to study the development, physiology, and evolutionary conservation of mammalian ovarian follicles.

## Significance

This paper improves knowledge of the somatic and germ cells of the developing mouse ovary that assemble into ovarian follicles, by determining cellular gene expression, and tracing lineage relationships. The study covers the last week of fetal development through the first five days of postnatal development. During this time, many critically important processes take place, including sex determination, follicle assembly, and the initial events of meiosis. We report expression differences between pregranulosa cells of wave 1 follicles that function at puberty and wave 2 follicles that sustain fertility. These studies illuminate ovarian somatic cells and provide a resource to study the development, physiology, and evolutionary conservation of mammalian ovarian follicle formation.

The basic outlines of somatic cell development in the mammalian fetal ovary are well-understood in rodents (1–4). In mouse, the coelomic epithelium (CE) forms on the ventral side of the mesonephros, beginning at about embryonic day 9.5 (E9.5), thickens, proliferates, and begins to express characteristic genes. Primordial germ cells (PGCs) reach the gonad about E10.5 where they proliferate mitotically to form cysts that partially fragment and aggregate together into cell “nests” surrounded by somatic cells (5–7). Prior to E12.5, surface epithelial progenitors engender multiple somatic cell types, including bipotential precursors of Sertoli or pregranulosa (PG) cells, interstitial cells, and steroid hormone producing cells (2, 8–11) which then support male or female gonad differentiation. Female development requires Wnt4/Rspo1/β-catenin signaling (12–15) and is aided and maintained by Foxl2 (16–19), but the key intercellular signals and target genes are not fully delineated. Eventually, two “waves” of follicles are produced; a first wave in the medullar region of the ovary develops rapidly while a second wave in the cortex produces primordial follicles that mostly arrest in order to sustain fertility throughout life (20, 21).

Two types of PG cells are involved in the production of medullar or primordial follicles. Bipotential progenitors are generated by mitotic activity in the surface epithelium during E11.5 and for some period afterward (8, 15). By E12.5, the key transcription factor Foxl2 (16) turns on in at least some bipotential-derived pregranulosa (BPG) cells, and lineage labeling shows that these cells give rise to granulosa cells exclusively within first wave follicles (8, 21). The second “epithelial-derived” pregranulosa (EPG) cell population begins production at least by E14.5, also from progenitors in the ovarian surface epithelium (22, 23). These cells express Lgr5 and eventually differentiate as granulosa cells on second wave follicles (8, 21–23). The cellular origins, division timing, and gene expression programs underlying both groups of PG cells need to be more precisely defined. Ultimately, the extent to which these cells control the different properties of the two follicular waves remains of interest.

Evolutionary conservation provides another potentially valuable source of insight into ovarian follicle development. In both mouse and *Drosophila*, primordial germ cells migrate to the gonadal primordium (24, 25), and oocytes differentiate within interconnected cysts of meiotic germ cells with the assistance of nurse-like cells that transfer organelles (26, 27). In both organisms, early PG cells generated from bipotential precursors express Wnts and signal to developing germ cells (15–19, 28, 29). In *Drosophila*, these early cells are termed “escort cells” (ECs) and arise from bipotential pupal gonadal “intermingled cells” (30). ECs are displaced from cysts at pachytene (31) by stem cell-derived follicle cells, which proliferate to form an epithelial follicular monolayer that mediates subsequent development to maturity (32, 33). Thus, in both species, two types of somatic support cells contact germ cells and contribute to folliculogenesis.

Additional insights into germ cell and follicle development have also come from genetic and physiological studies (4, 34). Genes responding to the meiotic inducer retinoic acid (RA) (35, 36), and to its key target Stra8, have been characterized (37, 38). Recently, mouse and other mammalian germ cell development has been further analyzed using single-cell RNA sequence (scRNAseq) analysis, especially in the male germline (39, 40) and in human ovaries (41). Early fetal mouse gonadal somatic cells of both sexes were purified and analyzed by scRNAseq to better understand sex differentiation (42). A powerful adjunct to scRNAseq for determining cellular relationships is lineage tracing (7) and the ability to reconstruct developmental trajectories (43).

Here, we analyze the developing mouse ovary using scRNAseq at seven time points between E11.5 and postembryonic day 5 (P5), involving a total of 52,542 cells. During E12.5 to P5, female germ cells express several thousand genes differentially as they pass through six meiotic stages, extending previous studies (37, 42, 44, 45). We also define genes expressed by epithelial progenitors and clarify the similar but distinct genetic programs of BPG and EPG cell progenitors (8, 21–23). Their differentially expressed genes are candidates to control the distinctive developmental programs of wave 1 and wave 2 follicles. These observations provide a strong basis for further studies of the development, physiology, and evolutionary conservation of mammalian ovarian follicles.

## Results

### Generation of a Single-Cell Atlas of Mouse Ovarian Follicle Development.

During mouse ovarian development, primordial germ cells arrive at the gonad (E10.5), undergo sex determination (E11.5), cycle mitotically to form germline cysts (E12.5), transition to meiosis (E14.5), progress to pachytene (E16.5), and undergo organelle transfer, cyst breakdown, oocyte differentiation, and germ cell turnover (E16.5 to P1), leading to primordial follicle formation by P5. Our experiments were designed to characterize cells involved in ovarian follicle development and generate a broader single-cell archive of ovarian cells for future studies. We sequenced 10 to 18 gonads/ovaries isolated at seven time points between E11.5 and P5.

**Fig. 1.**
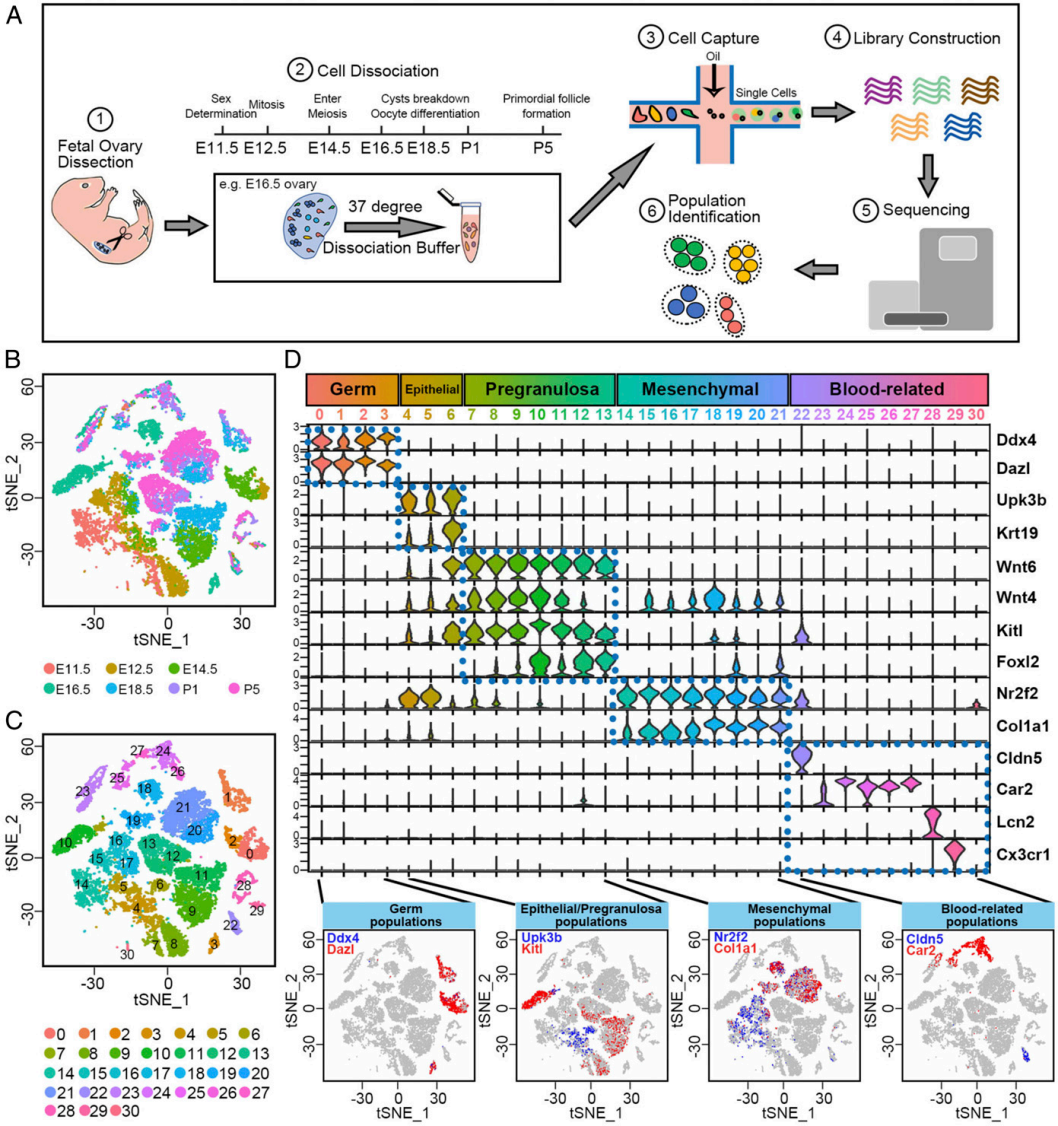
Single-cell transcriptome landscape of fetal ovarian development. (*A*) Schematic experimental workflow using the 10X Genomics Chromium platform followed by clustering using Seurat (43). (*B*) A 2D visualization of single-cell clusters using tSNE colored by developmental time from E11.5 to P5. (*C*) A 2D visualization of single-cell clusters using tSNE colored by 31 identified cell types/clusters (numbers). (*D*) Summary of marker gene expression in cell clusters: Clusters 0 to 30 were subdivided into five subclasses based on gene expression (dotted boxes). (*Bottom*) Violin plots show marker gene expression in each cluster. *y* axis scale: a normalized UMI-per-cell scale for each gene to facilitate display. The *Bottom* of *D* shows the expression of marker genes (red and blue) from each subclass.

At each time, after a meticulous dissection and trypsin incubation, ovaries were dissociated into single-cell suspensions. Dissociated cells were subsequently captured, loaded onto oil droplets, and used for complementary DNA (cDNA) library construction, deep sequencing, and cluster analysis (Fig. 1*A* and *Experimental Methods*). Expression information from 52,542 cells with an average of 2,700 different genes per cell was recovered based on 2.38 billion confidently mapped reads (*SI Appendix*, Table S1).

The datasets generated from the different time points were first analyzed jointly using the following strategy. Transcript counts were first normalized, log2 transformed, aligned, and integrated as described (43). Using a t-distributed stochastic neighbor embedding (tSNE) analysis, we arranged the integrated datasets in temporal order (Fig. 1*B*), and identified 31 clusters (Fig. 1*C*), which were classified within five major categories (Fig. 1*D*). These were germ cells (cluster 0 to 3) with Ddx4 and Dazl expression (46), epithelial cells (clusters 4 to 6) with Upk3b and Krt19 expression (47, 48), PG cells (clusters 7 to 13) with Wnt4, Wnt6, Kitl, and Foxl2 expression (49–51), mesenchymal cells (clusters 14 to 21) with Nr2f2 and Col1a1 expression (23, 52), and endothelial/blood-related cells (clusters 22 to 30) with Cldn5, Car2, Lcn2, and Cx3cr1 expression (53–56).

The cell groups were partially validated for selected germline, epithelial, PG, and mesenchymal clusters by staining developing ovaries with cluster markers (*SI Appendix*, Fig. S1). Costaining for Nr2f2, Foxl2, and Ddx4 confirmed that Nr2f2-expressing cells and Foxl2-expressing cells are mutually exclusive both in E12.5 and E18.5 ovaries (23). At E12.5, Foxl2 expression is detected in some somatic cells adjacent to germ cells, consistent with previous observations (16). The next step was to carry out a higher resolution analysis of the germline and somatic cells that participate in follicle formation.

### Fine Scale Analysis of the Germ Cell Meiotic Transcriptome.

**Fig. 2.**
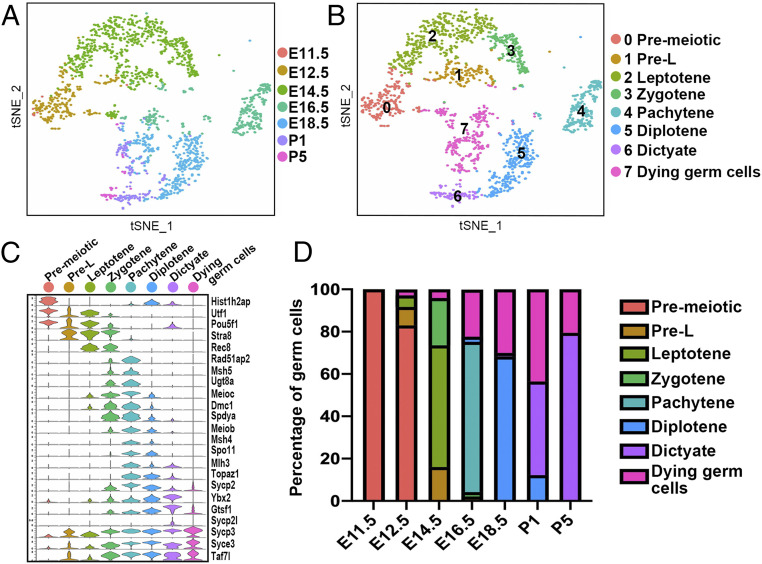
Dynamic gene expression patterns of mouse female germ cells. (*A*) Two-dimensional (2D) visualization of clusters from all germ cells using tSNE. Cells are colored by developmental stage from E11.5 to P5. (*B*) Two-dimensional visualization of integrated germ cell clusters using tSNE. Cells are colored by eight inferred developmental stages (see key for stage names). (*C*) Multiviolin plot of selected meiosis-related gene expression during the eight developmental stages. *y* axis scale: same as Fig. 1*D*. (*D*) Cell distribution among the eight stages at each time point.

We investigated the genetic program of germ cell development by reperforming tSNE analysis using only the germ cell clusters. Early stage germ cells (E11.5, E12.5, E14.5), which are completing mitotic divisions and just entering meiosis, map on the left and upper side of the tSNE plot whereas later stage germ cells (E16.5, E18.5, P1, and P5), which are traversing meiotic prophase and arresting as dictyate oocytes, localize in groups spaced largely in temporal order on the right and below (Fig. 2*A*). The only exceptions are small clusters of germ cells from each time point after E12.5 that scatter in the center, candidates for germ cells undergoing organelle transfer and programmed cell death. Although we analyzed between 4,100 and 11,400 cells per time point, the fraction of germ cells in the temporal groups meets expectation, with the most germ cells at E14.5, when mitotic proliferation has just finished. Thereafter, germ cell numbers fall as cysts break down, and ∼80% eventually turn over (7).

The germ population was computationally subdivided into eight transcriptionally distinct clusters whose expression patterns were found to match the temporal meiotic gene expression program (Fig. 2 *B* and *C* and Dataset S1). Premeiotic germ cells (cluster 0) express early germ cell markers (such as Utf1 and Pou5f1) and the mitotic marker (Hist1h2ap) (57). Preleptotene (Pre-L) cells (cluster 1) turn on the RA signaling pathway induced transcriptional regulator Stra8 (58). Leptotene cells (cluster 2) turn on Stra8 targets, such the cohesin subunit Rec8 supporting meiotic DNA replication (59). Meioc is also induced to extend meiotic prophase and prevent premature cell cycle arrest (37, 60). Leptotene cells up-regulate synaptonemal complex components, such as Sycp3, as homolog pairing progresses. Zygotene cells (cluster 3) express additional synaptonemal complex genes, including Sycp2 and Syce3, complete homolog alignment, and begin to up-regulate Spo11, Meiob, and Rad51ap2, which are required for meiotic recombination (61–63). In pachytene (cluster 4), high expression of these and other genes initiates double-stranded DNA (dsDNA) breaks, which utilize recombination and repair proteins, such as Msh4, Mlh3, and Ybx2, to further process and repair them into crossovers (64–66). Germ cells progress to diplotene (cluster 5) as they disassemble the synaptonemal complex, and wave 2 oocytes eventually arrest cycling and enter the dictyate state (cluster 6) found in primordial follicles. Dictyate follicles specifically express Sycp2l, which regulates the survival of primordial oocytes (67). A minority of cluster 6 cells correspond to wave 1 oocytes, which do not arrest but begin to develop as primary follicles. Overall, we profiled more than 2,500 genes whose expression varies substantially across the six meiotic substages (*SI Appendix*, Fig. S2 and Datasets S1 and S3).

The proportion of germ cells in each meiotic cluster varied largely as expected at each developmental time point (Fig. 2*D*). Most germ cells (83.0%) at E12.5 were classified as premeiotic. E14.5 germ cells were heterogeneous in stage (preleptotene, 16.1%; leptotene, 57.5%; and zygotene, 22.4%), reflecting differences in the time when PGC cells arrive at the gonad, as well as the anterior–posterior gradient of differentiation (37, 68). Most E16.5 cells were in pachytene (45) and had transitioned to diplotene by E18.5 (69). Interestingly, during E16.5 to P1, a variable but significant proportion of germ cells coclustered, displayed reduced unique molecular identifier (UMI) counts per cell, and were scored as nurse/dying cells (cluster 7). Because experiments are needed to understand how interconnected cyst cells fractionate at these stages, a detailed analysis of cluster 7 cells will be presented elsewhere. By P1, most oocytes are arrested at dictyate, but they continue to increase significantly in UMI counts through P5.

Immunofluorescence staining using Pou5f1 and Sycp3 antibodies in the early fetal ovaries validated the known spatial asynchrony of meiotic progression (*SI Appendix*, Fig. S1*C*). As expected, at E12.5, when the female germ cells undergo rapid division and form germline cysts, all germ cells exhibited strong staining with Pou5f1 but no staining with Sycp3. By E13.5, germ cells in the anterior region have begun to express Sycp3 while the germ cells at the posterior continue to show high Pou5f1 staining. It should be noted that germ cells located at the anterior surface still express Pou5f1 but not Sycp3 (*SI Appendix*, Fig. S1*C*, arrowheads), indicating that there is also a temporal difference in meiotic timing between the cortex and deeper layers. At E14.5, the percentage of Sycp3^+^ germ cells increased to 60.7%. These inverse trends continued until Pou5f1 expression completely disappeared at E16.5 (*SI Appendix*, Fig. S1 *C* and *D*). Thus, our analysis of germline gene expression identified the same meiotic stages across the multiple time points, despite temporal variation in when progenitors arrive at the gonad and spatial variation in germ cell development along the anterior–posterior ovarian axis.

### The Undifferentiated Ovarian Surface Epithelium Surrounds Bipotential Precursors in the E11.5 Gonad.

**Fig. 3.**
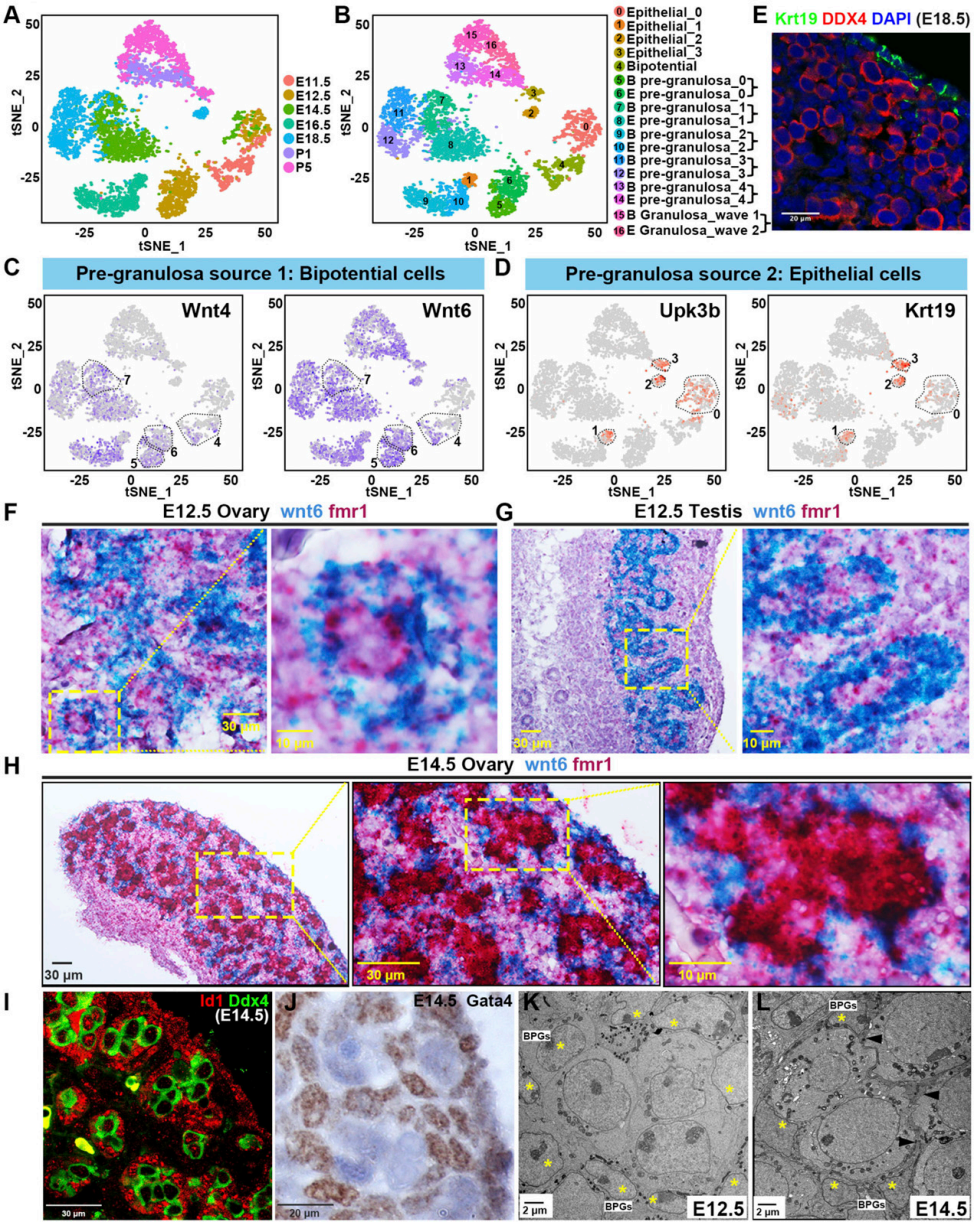
Identification and cellular localization of epithelial and PG cells. (*A*) A 2D visualization of clusters from “epithelial” and “pregranulosa” subgroup cells using tSNE. Cells are colored by embryonic time points from E11.5 to P5. (*B*) A 2D visualization of epithelial and PG cell clusters using tSNE colored by 17 identified cell groups. (*C*) Expression of Wnt4 and Wnt6 in PG cells; the color indicates level of expression. In *C* and *D*, dashed regions correspond to indicated cell clusters (*B*). (*D*) Expression of Upk3b and Krt19 of epithelial cells; the color indicates level of expression. (*E*) Cellular localization of Krt19 in E18.5 ovaries. Ovaries were stained for Krt19, and the germ cell marker DDX4 at E18.5 by immunofluorescence. (*F*–*H*) ISH analysis shows Wnt6 (blue) and Fmr1 (red) mRNA expression in the E12.5 ovary (*F*), E12.5 testis (*G*), and E14.5 ovary (*H*). Boxed regions are shown at higher magnification in the *Right*. (*I*) Cellular localization of Id1 in E14.5 ovaries. Ovaries were stained for Id1, and the germ cell marker DDX4 at E14.5 by immunofluorescence. (*J*) Cellular localization of Gata4 in E14.5 ovaries by immunohistochemistry. (*K*) Electron micrograph of E12.5 ovary showing part of a germline cyst (center) surrounded by BPG cells (yellow asterisks). (*L*) Electron micrograph of E14.5 ovary showing part of a germline cyst surrounded by BPG cells (yellow asterisks). Squamous membranes of BPG cells surrounding the germ cells are indicated by arrowheads. Scale bars are indicated.

We next investigated the somatic cells that interact with germ cells. Cells in just the ovarian epithelial and PG subgroups (Fig. 1*D*) were reanalyzed and displayed as before using tSNE. Four epithelial subclusters, one bipotential subcluster, and 12 PG subclusters were identified, which are shown by their time of development (Fig. 3*A*) or by cluster identity (Fig. 3*B*). The clusters will be referred to by cluster number (0 to 16) and, where possible, were also named by their deduced cellular state as described below. Cellular state was analyzed using previous knowledge, the expression status of genes relevant to BPG (Fig. 3*B*) and EPG cells, full transcriptomes (*SI Appendix*, Fig. S3 and Datasets S2 and S4) and by lineage tracing experiments. At E11.5, cells fell into only two categories: epithelial cells formed part of cluster 0 (Epithelial_0) while the PG precursors formed cluster 4 (Bipotential).

**Fig. 4.**
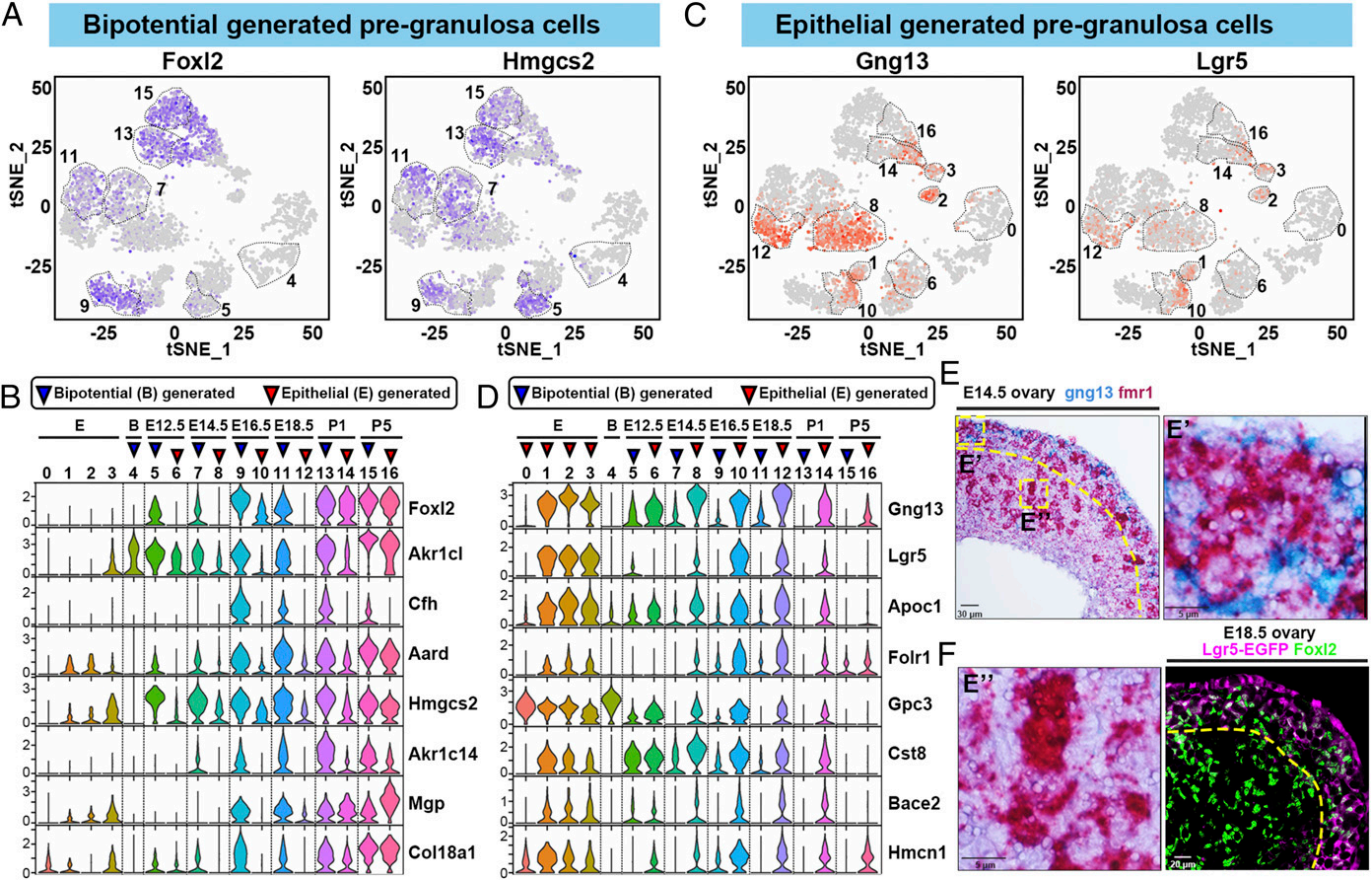
Distinct gene expression patterns of BPG and EPG cells. (*A*) Expression of Foxl2 and Hmgcs2 in epithelial and PG cell clusters; color intensity indicates level of expression. The thin dashed lines indicate bipotential and BPG clusters. (*B*) A multiviolin plot showing the relative expression of BPG marker genes (gene names at right) in cell clusters (numbers at top). Blue triangle: BP or BPG cluster; red triangle: EPG cluster. Epithelial clusters (E) and developmental times are also indicated. (*C*) Expression of Gng13 and Lgr5 in epithelial and PG cell clusters; color intensity indicates level of expression. The thin dashed lines indicate epithelial and EPG clusters. (*D*) A multiviolin plot showing the relative expression of EPG marker genes (gene names at right). The blue and red triangles indicate bipotential (B), epithelial (E), BPG, and EPG clusters. Developmental times are also indicated. (*E*) ISH of Gng13 (blue) and Fmr1 (red) mRNA in the E14.5 ovary. Gng13-expressing PG cells are observed in a cortical region (*E*′) but are absent from a medullar region (*E*″). (*F*) Cellular localization of Lgr5-GFP and Foxl2 in E18.5 ovaries by immunofluorescence. Curved dashed lines in *E* and *F* show the boundary of the cortical and medullar regions.

Many cluster 4 cells were verified as bipotential precursors based on 1) their presence at E11.5 prior to the completion of sex determination, 2) their nonmitotic signature (low Mki67 and Hist1h2ap but high Cdkn1b [encoding a p27 cell cycle kinase inhibitor]), 3) their expression (Fig. 3*C*) of the signaling molecules Wnt4 and Wnt6 (on average 10× higher than other clusters) but not markers of an epithelial origin (Fig. 3*D*), and 4) high Kitl, Rspo1, and Runx1 (17) expression. To investigate what cluster 4 cells give rise to after sex determination, we carried out whole mount double in situ hybridization (ISH) experiments on E12.5 ovaries using the cluster 4 marker Wnt6 messenger RNA (mRNA). In the E12.5 ovary, Wnt6-expressing cells were detected adjacent to germ cell nests (marked by Fmr1 expression) as expected for PG cells (Fig. 3*F*). Moreover, Wnt6-positive cells in the E12.5 testis encircled germ cells in developing cords, identifying them as Sertoli-like cells (Fig. 3*G*). Thus, Wnt6-positive E11.5 cluster 4 cells are likely to be bipotential precursors.

In contrast, cluster 0 cells likely correspond to the undifferentiated ovarian surface epithelium (OSE). Cluster 0 cells are found not only at E11.5, but also in ovaries from E12.5 and E14.5 (Fig. 3 *A* and *B*). Their presence at the ovarian surface is supported by strong ovarian surface labeling using anti-Krt19 antibodies (Fig. 3*E*), one of the epithelial marker genes they express (Fig. 3*D*). Unlike any of the granulosa clusters 4 to 16, cluster 0 cells also express high levels of Mki67 and other markers, suggesting they divide actively. In addition, cluster 0 cells highly express Lhx9 (1,380 mUMI per cell), a marker for undifferentiated ovarian cells (9). The coclustering of these epithelial cells from multiple time points spanning E11.5 to E14.5 represents another indicator that their state of differentiation is not changing much as they divide and produce progeny during this period (Fig. 3*A*).

### Both BPG and EPG Cells Are Produced during Early Fetal Ovarian Development.

Surprisingly, ovarian PG cells as arrayed by tSNE analysis contained paired PG clusters, one from the bipotential pathway and one from the epithelial pathway, at each developmental stage analyzed from E12.5 to P5 (Fig. 3*B*). At E12.5, these are clusters 5 and 6 (PG_0); at E14.5, clusters 7 and 8 (PG_1); at E16.5, clusters 9 and 10 (PG_2); at E18.5, clusters 11 and 12 (PG_3); at P1, clusters 13 and 14 (PG_4); and, at P5, granulosa cell clusters 15 and 16. Previously, studies using Foxl2 as a PG cell marker suggested that PG cells up to about E14.5 were likely to be of bipotential origin whereas EPG cells destined for cortical follicles are produced by E14.5 (8, 23), or possibly at a low level by E13.5 (22).

Closer examination of gene expression compelled our interpretation. E12.5 cluster 5 cells highly express genes characteristic of bipotential cells (Wnt4, Wnt6, Rspo1, Kitl, Runx1) and were derived from the E11.5 bipotential cells described above; hence, they are designated as bipotentially generated PG cells (BPG cells). Cluster 6 cells express much lower levels of bipotential cell genes, and we propose that they are recently generated by division of progenitors in the surface epithelium. Lhx9 expression is high in cluster 6 (1,250 mUMI per cell), only a little lower than in their presumed epithelial progenitors in cluster 0, while expression in cluster 5 is lower (737 mUMI per cell). Most importantly, Foxl2 expression is only 102 mUMI per cell in cluster 6 compared to 617 mUMI per cell in cluster 5. Foxl2 is a key gene defining PG cells and was relied on in many previous studies, but we show below that Foxl2 is only expressed at high levels in BPG cells, but not in EPG cells until the time of birth (P1). Ingressing cells from the OSE were reported previously at E12.5 that give rise to both Lgr5^+^ and Lgr5^−^ cells (8, 15). Cluster 6 cells express only low average levels of Lgr5 (166 mUMI per cell) as do cluster 0 cells (89 mUMI per cell). However, these progenitors may make up only a small proportion of the OSE (16), and we show below that EPG cells can be lineage-labeled with Lgr5-cre as early as E13.5.

### By E14.5, Mitotic Surface Epithelial Cells Give Rise to PG Cells Expressing Pathway Markers.

By E14.5, the PG cell subclusters from the two major pathways, cluster 7 and 8, become more distinct. Cluster 7 resembles other BPG cells in expressing bipotential genes, as well as BMP2, Id1, and GATA4. Double ISH with Wnt6 and Fmr1 at E14.5 showed that cluster 7 cells remain tightly associated with germ cell cysts (Fig. 3*H*). Immunofluorescence studies showed that cells positive for Id1 tightly wrap germline cysts/nests in the E14.5 ovary (Fig. 3*I*). Staining E14.5 ovaries with anti-GATA4 antibodies gave an identical pattern (Fig. 3*J*). Thus, gene expression pattern and tissue location justify designating cluster 7 as BPG_1. EMs at E14.5 suggest many PG cells have taken on a squamous morphology by this time (compare Fig. 3 *K* and *L*). Compared to cluster 5, cluster 7 cells express Foxl2 in a larger fraction of cells (Fig. 4*A*), and cluster 7 cells expressed significantly higher levels of multiple other genes also expressed by other BPG cells, but not by PG cluster 8 (Fig. 4*B*). These included the ketogenic gene Hmgcs2 (70), the hydroxysteroid dehydrogenase gene Ark1c14 (71), and multiple others (Fig. 4*B*).

The second major population of E14.5 PG cells, cluster 8, showed clear evidence of a recent epithelial origin distinct from BPG cells. Cluster 8 cells strongly express Gng13 (Fig. 4*C*), a gene known to be restricted to expression at the ovarian surface during sexual differentiation (72), while some contained high levels of Lgr5 (Fig. 4*C*), a marker of EPG cells (8, 21–23). Thus, cluster 8 cells appear to have arisen by division of undifferentiated epithelial cells, the only source of dividing cells in the surface epithelium, followed by invasion into the ovarian cortex. E14.5 was the only time when the EPG pregranulosa cluster (cluster 8) contained more cells than the cotemporal BPG cluster (1,419 compared to 646 cells).

Comparing the transcriptomes of clusters 7 and 8 revealed several other genes that are expressed differentially in cluster 8 (Fig. 4*D*). To show that cluster 8 cells correspond to ingressing PG cells, we carried out double ISH at E14.5 with Gng13, which is expressed on average at 2,500 mUMI per cell in cluster 8, compared to 585 mUMI per cell in cluster 7. Fmr1 was used to label germ cells. Somatic cells positive for Gng13 were readily observed surrounding germ cells (Fig. 4 *E* and *E'*). However, those germ cells with a Gng13-positive cell layer were located mostly in the outer region of the ovarian cortex (Fig. 4*E*). Germ cells located in the medulla lacked somatic cells expressing Gng13 at E14.5 (Fig. 4 *E* and *E''*). These data suggest that the cortical Gng13^+^ PG cells represent surface epithelium-derived cells that have migrated inward and surrounded cysts within the outer ovarian cortex, but few if any of these cells have reached the inner cortex or medulla region. The same distribution was reported for Lgr5-expressing cells at E14.5 (23).

### Identification of BPG and EPG Cells in Ovaries from E16.5 to P1.

At E16.5 and after, the nondividing PG cell clusters defined by scRNAseq in Fig. 3*B* continued their orderly progression, with cluster 9 representing BPG cells and cluster 10 representing EPG cells based on gene expression, as before. This suggested that the EPG and BPG pathways of PG cell production evident by E14.5 simply continue their programs of development for the remainder of follicle progression up to P5 when primordial follicles are largely complete. One difference at E16.5 was that the epithelial cells were no longer classed with cluster 0. They had begun to differentiate as indicated by a new location next to E16.5 EPG cells in cluster 1 (Fig. 3*D* and *SI Appendix*, Fig. S3). Cluster 1 remained small in size, and mitotic activity as measured by Mki67 levels declined sharply from 809 mUMI per cell in cluster 0 to 69 mUMI per cell at E16.5 (cluster 1) and 15 mUMI per cell at E18.5 (cluster 2). A spike in activity to 95 mUMI per cell was seen at P1 before falling again at P5. These changes suggest that differentiating epithelial surface cells strongly reduce their mitotic activity and significantly curtail new PG cell production by E16.5.

The presence of a BPG cluster paired with an EPG cluster continued at E18.5 (clusters 11 and 12) and P1 (clusters 13 and 14), respectively. Gene expression analyses comparing differential gene expression during PG cell differentiation found many similarities between the pathways, but also a significant number of differentially expressed genes at each time point (*SI Appendix*, Figs. S3 and S4 and Dataset S2). Foxl2 expression was 2.7 times higher in the BPG cluster compared to the EPG at E14.5, 2.56 times higher at E16.5, but only 1.25 times higher at P1 and 1.06 times at P5 due to increased Foxl2 expression in EPG cells at P1. In contrast, throughout all stages of differentiation, Lgr5 is preferentially expressed in EPG cells. Lgr5 levels are 2.88 times higher in EPG cells compared to BPG cells at E14.5, 8.0 times higher at E16.5, 7.69 times higher at E18.5, and more than 15 times higher at P1 and P5. Immunofluorescence staining at E18.5 of Lgr5-EGFP ovaries showed that Lgr5-positive PG cells were almost entirely cortical while Foxl2-labeled both medullar follicles and some cortical follicles (Fig. 4*F*). Overall, the BPG and EPG pathways expressed 1,700 to 2,300 distinctive genes, 1,400 to 1,800 of which were common, with about 100 to 250 genes being significantly enriched in one pathway or the other at any given time (*SI Appendix*, Fig. S4 and Datasets S2 and S5).

### Lineage Tracing Shows that BPG Cells Are Largely Displaced from the Cortex by Invading EPG Cells.

**Fig. 5.**
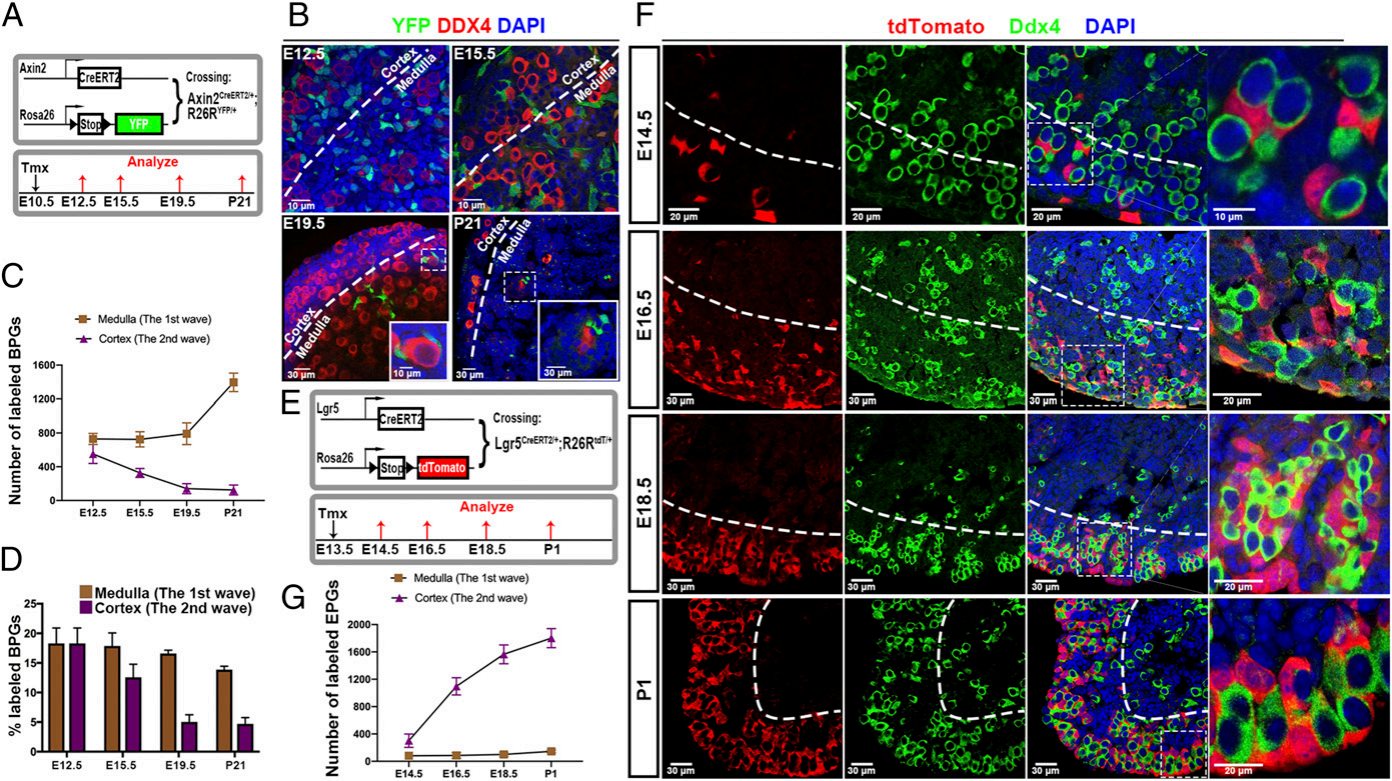
Lineage tracing of BPG cell replacement by cortical EPG cells. (*A*) Strategy for lineage-tracing bipotential cells. Mice containing Axin2^CreERT2/+^ mice and R26R^YFP/YFP^ received tamoxifen (Tmx) at E10.5, and ovaries were analyzed at E12.5, E15.5, E19.5, and P21. (*B*) YFP-marked cells (green) are seen adjacent to germ cells (red) in the medulla and in the cortex until E19.5. Boxed regions correspond to *Insets*. (*C*) Quantitation of YFP-labeled BPG cells in the cortex and medulla at each time point shown in *B*. (*D*) Percentage of cyst/follicle-associated PG cells labeled in the medulla and cortex at each time. (*E*) Strategy for lineage tracing EPG cells. Mice containing Lgr5^CreERT2/+^ and R26R^tdT/tdT^ reporter received Tmx at E13.5, and ovaries were analyzed at E14.5, E16.5, E18.5, and P1. (*F*) tdTomato-positive EPG cells (red) were sparse at E14.5 but associated with germ cells (green). Cortical tdTomato^+^ cells increased significantly at E16.5, E18.5, and P1, but very few entered the medullar region. Boxed regions correspond to *Right* *Column*. (*G*). Dashed lines in B and F show the boundary of the cortical and medullar regions.

The two pathways were investigated further using lineage marking. To mark bipotential cells as early as possible, we used the Wnt-responsive gene Axin2 (which is expressed in bipotential cells at E11.5). Axin2^CreERT2/+^ mice were crossed to Rosa26-YFP reporter mice, and pregnant females received tamoxifen (Tmx) at E10.5. Ovaries were analyzed at E12.5, E15.5, E19.5, and P21 (Fig. 5*A*). The fact that cells labeled in this manner only contributed to wave 1 follicles confirmed that only bipotential cells had been labeled. At E12.5, BPG cells were extensively labeled in both the cortex and medulla of the ovary where they contact virtually all germ cell cysts at a similar density (Fig. 5*B*). To aid visualization, the approximate boundary between the cortical and medullary regions (parallel to the ovarian surface) in the sections are indicated by a dashed line since this can be judged best in the context of low magnification. While it is not possible to precisely distinguish a boundary between these zones, especially in young ovaries, the conclusions of our experiment do not depend on the exact placement of this boundary beneath the surface.

While, initially, the distribution of labeled BPG cells appeared uniform, by E15.5, the number of labeled cells in the cortical (but not the medullar) region was reduced in absolute numbers (Fig. 5*C*). At E19.5 and again at P21, in contrast, labeled cells were found exclusively in the medullar region, indicating that cortical BPG cells had been fully displaced by EPG cells rather than undergoing transdifferentiation (Fig. 5*B*). Fig. 5*C* summarizes the loss of lineage-marked BPG cells from the cortex and their retention in the medulla. To compensate for ovarian and follicle growth, which dilutes the number of labeled cells per unit area, we also calculated the percentage of somatic cells associated with each cyst/follicle that were bipotential lineage-marked over time (Fig. 5*D*). In the cortex, the percentage of BPG-marked cells per cyst or follicle fell sharply from 18 to 4%, a background level that results from the ambiguous cortical/medullar boundary or possibly from a small amount of BPG cell survival. In contrast, the percentage of BPG-marked cells per cyst or follicle in the medulla decreased only slightly, from 18% at E12.5 to 15% at P21 (Fig. 5*D*). The increase in cell number observed at P21 is likely due to the mitotic growth of wave 1 granulosa cells in primary follicles.

### Kinetics of Lgr5^+^ EPG Replacement of Cortical but Not Medullar BPG Cells.

We further investigated Lgr5^+^ EPG association with cortical follicles using lineage marking. Lgr5^CreERT2/+^ mice were crossed to Rosa26-tdTomato reporter mice, and we administered Tmx to pregnant females at E13.5. Ovaries from offspring at 14.5, 16.5, 18.5, and P1 were then collected and analyzed (Fig. 5*E*). Since dividing Lgr5^+^ cells are confined to the ovarian surface, this protocol is expected to label newly generated Lgr5^+^ EPG cells and reveal their subsequent behavior.

When we analyzed embryos at E14.5 whose ovarian cells had been labeled in this manner at E13.5, several results were clear (Fig. 5 *F* and *G*). The great majority of the tdTomato^+^ (tdT^+^) cells clonally derived from Lgr5^+^ EPG cells labeled at E13.5 were still found near the ovarian surface although some cells had migrated inward to near the estimated position of the cortical/medullar boundary (Fig. 5*F*, dashed line). By E16.5, the number of tdTomato^+^ cells had increased and spread throughout the cortical region while the small number of medullar cells showed little change. By E18.5 and P1, cortical germ cells were almost entirely surrounded by tdTomato^+^ cells (Fig. 5 *F*, *Right Column*). The few remaining unlabeled cells around cortical germ cells were probably Lgr5^+^ cells that escaped lineage marking since marked BPG cells were gone by E19.5 (Fig. 5*C*). In contrast, tdT^+^ cells were only seen at very low levels in the medullary region, showing that EPG cells never take up residence in the medulla in significant numbers (Fig. 5*G*).

**Fig. 6.**
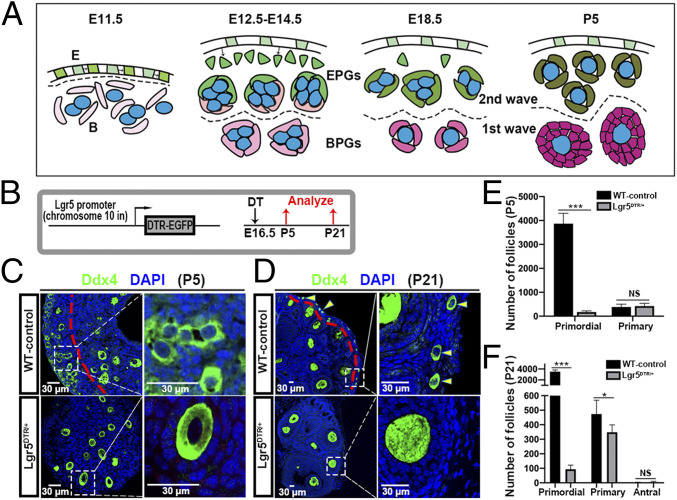
Lgr5-expressing cell ablation impairs second wave follicle formation. (*A*) Model of Epithelial progenitor cells (E), Bipotential cells (B), BPG cells (pink), and EPG cells (green) in forming first wave 1 and second wave follicles. Germ cells (blue). Developmental times are indicated. Dashed line separates ovarian cortex (above) from the medulla. (*B*) Experimental strategy to ablate Lgr5-expressing cells using the Lgr5-DTR-EGFP mouse model. (*C*) Histological analysis of ovaries from wild-type (WT) mice and Lgr5^DTR/+^ animals at P5. Boxed regions are magnified in the *Right*. (*D*) Histological analysis of ovaries from WT mice and Lgr5^DTR/+^ animals at P21. Yellow arrowheads correspond to primordial follicles. (*E*) Quantification of primordial (wave 2) and primary (wave 1) follicles at P5 after DT administration at E16.5. (*F*) Quantification of primordial (wave 2), primary (wave 1), and antral (wave 1) follicles at P21 after DT administration at E16.5. NS, not significant. *P < 0.05, ***P < 0.001 (*t* test). (Scale bars: 30 μm.)

We used this same marking system to examine the fate of Lgr5^+^ daughters labeled in the OSE at P1 (*SI Appendix*, Fig. S5*A*). This allowed us to address whether any continuing epithelial progenitor division at the ovarian surface produces new EPG cells after birth. Although tdT-labeled cells could readily be seen at both P2 and P6 at the ovarian surface, none of these cells migrated into the ovarian cortex (*SI Appendix*, Fig. S5 *B* and *C*). Thus, EPG cells are no longer generated in detectable numbers after birth at P1. A model summarizing somatic cell behavior during first and second wave follicle formation is shown in Fig. 6*A*.

### Depletion Experiments Confirm that Lgr5^+^ Cells Give Rise Mainly to the Second Wave of Follicles.

We used the Lgr5-DTR-EGFP mice (73) to ablate Lgr5^+^ cells during fetal follicle development by treatment with diphtheria toxin (DT) to test the prediction of our studies that only second wave (cortical) follicles should be affected (Fig. 6*B*). In control mice which were treated with DT at E16.5, but lacked the transgene, a robust population of nearly 4,000 primordial follicles was observed at P5 in the cortical region, along with about 400 rapidly developing first wave medullary primary follicles (Fig. 6 *C* and *E*). In contrast, pregnant females carrying the construct that were DT-treated at E16.5 and examined at P5 contained less than 200 primordial follicles (<5% of controls) while the number of wave one primary follicles was unchanged (Fig. 6 *C* and *E*). Similarly, at P21, DT-treated controls contained more than 3,500 cortically located primordial follicles (Fig. 6*D*, arrowheads) and 450 medullar primary follicles (Fig. 6 *D* and *F*). By comparison, Lgr5^DTR/+^ animals treated with DT retained less than 100 primordial follicles or about 3% of controls (Fig. 6 *D* and *F*). The number of wave one primary follicles was reduced to about 300 (67% of controls). These results strongly support the conclusions of our previous experiments that EPG cells nourish the second wave of follicles, but that first wave follicles retain BPG cells that develop in parallel with EPG cells in the cortex and support medullar follicle development without any significant contribution from surface-derived Lgr5^+^ PG cells.

## Discussion

### A Resource for Understanding Early Mouse Oogenesis.

We gained insight into the cellular and genetic foundations of mouse oogenesis, as well as a valuable resource for future studies, by analyzing single-cell transcriptomes from more than 52,500 cells isolated from E11.5 to P5 gonads/ovaries. We identified more than 2,500 genes whose expression changes as ovarian germ cells pass through six stages of meiotic prophase (*SI Appendix*, Fig. S2 and Dataset S3). Knowing the transcriptomes of these cells will greatly aid comparisons to meiosis in male gametes (40), and to female gametogenesis in other organisms (41, 74). Particularly exciting are the opportunities germline gene expression programs provide to better understand the important changes in the cytoplasm of meiotic cells. These include major alterations in cytoskeletal polarity and in the remodeling and movement of organelles, including endoplasmic reticulum, mitochondria, Golgi, and centrosomes. These programmed changes begin with sex determination and are accomplished in partnership with closely associated somatic cells. Although less studied, germ cell cytoplasmic modifications may be as strongly conserved as meiotic hallmarks, such as recombination, chromosome segregation, and suppression of transposon activity and meiotic drive.

### Two Populations of Granulosa Cells both Have Early Roots in the Gonad.

The acquisition of epithelial supporting cells plays a central role in gonad development (1–4, 8, 21–23). We used the power of scRNAseq to zero in on how epithelial populations change during fetal oogenesis, without confusion from mesodermal, endothelial, and germ cells or the perturbations of cell sorting. Cell groupings and their gene expression argued that PG cell formation from bipotential and epithelial progenitors is not strictly sequential, but overlaps in time and occurs in a similar manner. As expected, bipotential support cells were first detected at E11.5 where they are produced along with other somatic cells by divisions within the CE and migration into the gonad (8). In the E12.5 ovary, the derivatives of these cells are present and associating with germ cells. Unexpectedly, our data indicated that epithelial pathway PG cells are already likely to be present as well. Because we have no unique marker for these cells at E12.5, it is not clear if they are still located in the OSE near their progenitors, or if they have already entered the ovary and are generating cysts with hybrid PG cells of both pathway types. Gene expression suggested that looking for E12.5 cells expressing high Gpc3 and low Foxl2 might allow such identification in the future.

There is inherent uncertainty in relying on gene expression data alone to deduce developmental events. However, lineage-tracing studies support these conclusions. We labeled BPG precursors at E10.5 using Axin2-cre and observed their widespread distribution throughout the E12.5 ovary. We and others (23) labeled the EPG cells using Lgr5-cre at E13.5 and mapped them in the cortical region by E14.5, consistent with Lgr5-EGFP staining of OSE and subcortical PG cells at this time (22). How long new PG cells continued to be generated by these divisions is less well determined. While PG cells in the wave 2 pathway have been labeled with Lgr5-cre at E13.5, E14.5, E16.5, E17.5, and P1 (8, 22, 23), whether PG precursors in the OSE continue to divide and ingress at these times was not always determined. We found, at P1, that surface cells were readily labeled, but no newly labeled cells entered the ovary (*SI Appendix*, Fig. S5), suggesting that generation of PG cells from the OSE may be limited to the fetal gonad, at least in the absence of damage. The average levels of Mki67 in OSE fell 12-fold between E14.5 and E16.5, and 53-fold by E18.5 (Dataset S2), consistent with declining cell production.

### Foxl2 Is Differentially Expressed in BPG and EPG Cells.

A major difference in the two pathways is the preferential expression of Foxl2 in fetal BPG cells compared to EPG cells. There is sixfold greater average Foxl2 expression in cluster 5 BPG cells compared to cluster 6 EPG cells, and the differential remains around threefold from E14.5 to E18.5. Foxl2 expression only becomes nearly equal at P1 (1.25×), in agreement with previous study (16). Lineage labeling using Foxl2-cre at E12.5, E14.5, and E16.5 only marked cells that become part of wave 1 follicles (8, 21). The failure of Foxl2-induced lineage marking to include epithelial precursors prior to birth (21) may be a threshold effect. The absolute level of Foxl2 in group 5 BPG cells of 0.62 Umi per cell is not achieved in EPG cells until P1 when it reaches 1.1 Umi per cell in cluster 14. It is unclear what effects different levels of Foxl2 expression have on developing PG cells. Higher levels of Foxl2 may allow BPG cells to carry out unique roles during fetal development, such as hormone signaling, that are important for maintaining female differentiation. The differential gene expression identified here between BPG cells and EPG cells may be at least partly dependent on these differences in Foxl2 expression.

Higher resolution study of the fetal OSE is likely to provide further insights. A simple model postulates that both BPG cells and EPG cells are produced in the OSE but by distinct progenitors. There is evidence that the OSE is heterogeneous, and that only a subset of its cells express Lgr5 (22, 23). Wnt4/Rspo1-mediated signaling from BPG cells may stimulate Lgr5-expressing EPG progenitors (75), suggesting that progenitors and/or progeny of these pathways, interacting closely in time and space, act to homeostatically coregulate PG cell production and ovary development. Mutations influencing the BPG and EPG cellular composition might exert their effects by changing these interactions.

### Two Pregranulosa Cell Pathways Support Two Different Follicle Classes.

The timing and duration of fertility are critical parameters in the reproductive strategy of every species. Both wave 1 and wave 2 follicles play important roles in setting these values for mammals such as the mouse. The development of wave 1 follicles is accelerated to generate the initial finished follicles that determine the potential onset for fertility (21). In contrast, the number of wave 2 follicles and their stability over time control the duration of fertility. Our data showed that largely separate populations of PG cells differentiate in medullar and cortical follicles, suggesting that BPG cells assist rapid direct follicle development, and that EPG cells reinforce germ cell quiescence, stability, and regulated activation.

Genes differentially expressed in the two PG cell pathways are candidates for regulating follicular development. The rate of *Drosophila* ovarian follicle development can be strongly modulated by nutrition, as reflected in insulin signaling (76), and mammalian follicular growth is additionally influenced by activin/inhibin and steroid signaling (77, 78). BPG cells consistently express more of the androgen-degrading enzyme 3α-hydroxysteroid dehydrogenase encoded by Akr1c14 (71), which may help to sustain female development and promote direct development of wave 1 follicles. Akr1c14 is expressed 8 to 20 times higher in BPG cells than in EPG cells from at least E12.5 to E18.5. Several other BPG-enriched genes—including Hsd17b7, encoding an enzyme involved in cholesterol and steroid hormone biosynthesis (79); Hsd17b1, a gene that can masculinize female mice if overexpressed (80); and Aard, a gene that is expressed in Sertoli cells in developing testis (81)—may maintain a female hormonal environment and one conducive to primary follicle development in the medulla. Indeed, by P5, the BPG cluster selectively expresses Amh, Esr2, and Nr5a2, genes expressed during primary follicle development, as some medullar follicles have already begun primary follicle development.

In contrast, EPG cells consistently expressed more Lgr5 (82), and more Aldh1a2 after P1 (23). Aldh1a2 encodes an RA synthase, and RA stimulates gonadal cells to produce Foxl2, Esr2, and Wnt4 (36). While the role of RA in meiosis initiation is not fully understood (83), Aldh1a2 expression and RA production in EPG cells might promote the Foxl2 up-regulation that occurs around birth in EPG cells in cortical follicles. Gpc3 encodes a glypican found in the extracellular matrix. A similar protein, encoded by the *Drosophila dally* gene, is a major regulator spatially restricting proliferative signals from the germline stem cell niche (84). Gpc3 might play a role in preparing cortical follicles for quiescence, by restricting the access of growth factors. Other potentially relevant genes preferentially expressed in EPG cells are shown in Fig. 4*D* (also, see *SI Appendix*, Fig. S4).

### Mouse BPG Cells Resemble *Drosophila* ECs.

Mouse BPG cells show several similarities to *Drosophila* ECs, the cells that interact with developing germline cysts during early steps in oogenesis. Like BPG cells, *Drosophila* ECs arise from the bipotential precursors known as intermingled cells (30). ECs surround and signal to germ cells during cyst formation, premeiotic S phase, and during the leptotene through pachytene stages of meiosis, all analogous to events in the mouse ovary that occur between E11.5 and E18.5. If germ cells at these stages are ablated in adult *Drosophila* ovaries, ECs turn over (85). Wnt signaling from ECs influences other somatic cells and is important for ongoing germ cell development (86–89). In addition, disrupting EC gap junctions (90) or steroid signaling (91, 92) perturbs germ cell development. Another interesting similarity between BPG cells and *Drosophila* ECs is that both interact with early follicles but are subsequently replaced by a second somatic cell type. The finding that somatic cells on most germline cysts are replaced by an independent epithelial cell population at an analogous developmental stage in both *Drosophila* and mouse ovaries suggests that cellular succession is functionally important and has been conserved in evolution. The transcriptomes of mouse BPG cells (Dataset S2) will make it easier to compare the early stages of ovarian follicle formation in mammals and invertebrates (29, 93, 94).

### What Determines the Relative Size of the Wave 1 and Wave 2 Follicle Populations?

Our experiments provide evidence that the two populations of PG cells are similar but not identical. They both derive from divisions of epithelial progenitors spread over much of the gonadal surface followed by inward migration over a similar period of time, encompassing E11.5 to at least E14.5. It is already known that multiple types of cells are produced in this manner in the E11.5 gonad and that the fates of ingressing cells change abruptly with time (8, 11). Our studies will aid in understanding how the genes expressed in these progenitors, their daughters, and other ovarian cells regulate the establishment, relative size, and behavior of wave 1 and wave 2 ovarian follicles. Such knowledge, and the broader resources described here will assist in advancing insight into many other important aspects of ovarian function.

## Experimental Methods

### Animals.

Mouse experiments in this study were performed in accordance with protocols approved by the Institutional Animal Care and Use Committee of the Carnegie Institution of Washington. Lgr5-DTR-EGFP mice were obtained from Genentech (South San Francisco, CA). R26R-tdTomato mice were obtained from the laboratory of Chen-Ming Fan, Carnegie Institution for Science, Baltimore, MD. Lgr5-CreERT2 mice (008875), Axin2-CreERT2 mice (018867), and R26R-EYFP reporter mice (006148) were acquired from The Jackson Laboratory.

### Labeling and Tracing Experiments.

The R26R-tdTomato females were crossed with the Lgr5-CreERT2 males; those with a vaginal plug were considered as E0.5. The pregnant females at E13.5 or newborn pups at P1 were given a single intraperitoneal (i.p.) injection of Tmx (10 mg/mL in corn oil) at 1 mg per 25 g of body weight. The R26R-EYFP females were crossed with the Axin2-CreERT2 males, and the pregnant females at E10.5 were injected i.p. with Tmx at 0.2 mg per 25 g of body weight.

### DT Injection.

Pregnant mice (E16.5) were injected i.p. with 10 μg/kg DT solution in phosphate-buffered saline (PBS).

### Immunofluorescence and Immunohistochemistry.

Ovaries were fixed in cold 4% paraformaldehyde overnight, incubated sequentially in 10% and 20% sucrose in PBS overnight, embedded in OCT (optimal cutting temperature medium), and stored at −80 °C until cryosectioning. After high-temperature antigen retrieval with 0.01% sodium citrate buffer (pH 6.0), the frozen sections (10 μm) were blocked with 10% normal donkey serum for 30 min and then incubated with primary antibodies overnight at 4 °C. The primary antibodies used are presented in *SI Appendix*, Table S2. For immunofluorescence, the sections were washed with wash buffer and incubated with the appropriate Alexa-Fluor–conjugated secondary antibodies (1:200; Invitrogen) at room temperature for 2 h. After staining with DAPI, samples were analyzed using confocal microscopy (Leica SP5). For immunohistochemistry, the slides were incubated with avidin-conjugated secondary antibodies (ab64264; Abcam) before being exposed to diaminobenzidine (DAB) (ab64264, Abcam) for 1 min and then counterstained with hematoxylin.

### ISH.

Tissue samples were fixed in neutral buffered formalin (NBF) (10%) at room temperature for 24 h, embedded in paraffin, and sectioned to a thickness of 5 μm. Tissue was pretreated with boiling 1× Target Retrieval followed by Protease III at 40 °C for 30 min. After pretreatment, the samples were hybridized with probes against mouse *Wnt6* (401111), *Lgr5* (312171-C2), *Gng13* (462531), and *Fmr1* (496391-C2) using RNAscope 2.5 HD Duplex Assay (322430; ACDBio). Signal was detected by two different chromogenic substrates (HRP-C1-Green and AP-C2-Red). Finally, slides were counterstained with hematoxylin and covered with mounting medium.

### Tissue Dissociation and Single-Cell Library Preparation.

Perinatal ovaries were dissected and placed in 1× PBS on ice and then dissociated into single cells using 0.25% Trypsin at 37 °C with pipet trituration at intervals. E11.5 and E12.5 ovaries were dissociated for 20 min, E14.5 and E16.5 ovaries for 40 min, E18.5 ovaries for 1h, and P1 and P5 ovaries for 80 min. After being neutralized with 10% fetal bovine serum, dissociated cells were passed through 70-μm and 30-μm cell strainers, separately. Approximately 10,000 live cells were loaded per sample onto the 10X Genomics Chromium Single Cell system using the v2 chemistry per the manufacturer’s instructions (95). Single-cell RNA capture and library preparations were performed according to the manufacturer’s instructions. Sample libraries were sequenced on the NextSeq 500 (Illumina). Sequencing output was processed through the Cell Ranger 3.1.0 mkfastq and count pipelines using default parameters. Use of version 3.1 resulted in the recovery of more cells, including those with low Umi values, such as cluster 7 germ cells. Reads were quantified using the mouse reference index provided by 10X Genomics (refdata-cellranger-mm10v3.0).

### Cell Identification and Clustering Analysis.

Package “Seurat” v2.3.4 (https://satijalab.org) (43) was used to analyze the scRNAseq data. The count data produced by Cell Ranger pipelines was a UMI count matrix with genes as rows and cells as columns. The value means the number of UMIs that was detected. The count data were read and transformed into Seurat object using the *Read10X* and *CreateSeuratObject* function, separately. Cells with too few reads were filtered out using the *FilterCells* function (subset.names = “nGene”, low.thresholds = 200). Filtered count matrices for each library (E11.5, E12.5, E14.5, E16.5, E18.5, P1, and P5) were Log-normalized, scaled, and merged to an integrated dataset through the *NormalizeData*, *ScaleData*, and *MergeSeurat* functions. After detecting the variable genes (x.low.cutoff = 0.0125, x.high.cutoff = 5, y.cutoff = 0.5), cell clusters were determined and identified based on the SNN algorithm (reduction.type = “pca”, dims.use = 1:10, resolution = 0.6), and visualized through dimensionality reduction by the *RunTSNE* function. For reanalyzing germ population or PG population, clusters with unique expression of germ cell markers or granulosa cell markers were extracted from the integrated dataset by the *SubsetData* function. The isolated cluster was divided into several subclusters after a series of normalization, scale, and dimensionality reductions.

### Data or Gene Profiles Visualization.

Violin plots were used to visualize the specific gene expression distributions in each cluster through the *VlnPlot* function within Seurat. The *y* axis of a violin plot represents the data slot of each Seurat object (object@data) which stores normalized and log-transformed single-cell expression. The data slot maintains the relative abundance levels of all genes and contains only zeros or positive values. The data slot was also used to visualize gene expressions in low-dimensional space through the *FeaturePlot* function. Unlike in the violin plot and feature plot, the heat maps (*SI Appendix*, Figs. S2 and S3) use the scale.data slot (object@scale.data), but not data slot, as the input source and was performed through the *DoHeatmap* function. To make our dataset more accessible and usable, the data slot of each germ cell cluster (Fig. 2*C*) or PG cluster (Fig. 3*B*) was extracted. The values (Datasets S1 and S2) represent the mean milliUMI (mUMI) per cell after row sum and average of the data slot in each cluster (object@data sums and divided by cell numbers).

## Supplementary Material

Supplementary File

Supplementary File

Supplementary File

Supplementary File

Supplementary File

Supplementary File

## Data Availability

Sequence data have been deposited in the GEO database (GSE136441) (96).
