# Supplementary material for: Two distinct pathways of pregranulosa cell differentiation support follicle formation in the mouse ovary
Source: Proc Natl Acad Sci U S A. 2020 Aug 5;117(33):20015–26. doi: 10.1073/pnas.2005570117 (PMC7443898; doi:10.1073/pnas.2005570117)
Supplement: Supplementary File [file pnas.2005570117.sapp.pdf]

Supplemental Information Appendix

Supplemental Tables

Table S1. Single cell RNA seq

Stage	Number of cells	UMI/cell	Genes/cell	Number of ovaries	Reads xE06	Well aligned
E11.5	8,293	10,036	3,143	18	513	461
E12.5	4,744	10,984	3,214	16	210	181
E14.5	11,417	6,251	2,483	18	510	457
E16.5	11,042	10,712	3,493	14	558	490
E18.5	4,118	6,704	2,426	14	472	371
P1	6,409	6,995	2,229	14	258	217
P5	6,519	6,512	2,245	10	247	208

UMI/cell and genes/cell represent averages across the all cell types, and may not be comparable to values for individual cell groups in Datasets S1-S2.

Table S2. Materials list

**Reagents or Antibodies**	**Producer or Source**	**Catalog# and Lot #**
**Antibodies**		
anti-Ddx4	Abcam	Cat#Ab13840; Lot#GR294410-1
anti-Ddx4	Abcam	Cat#Ab27591; Lot#GR290112-3
anti-YFP	Aves Labs	Cat#GFP-1020; Lot#GFP697986
anti-Nr2f2	R&D Systems	Cat#PP-H7147-00; Lot#A-2
anti-Sycp3	Santa Cruz	Cat#Sc-74568
anti-Gata4	Santa Cruz	Cat#Sc-25310; Lot#H0613
anti-Foxl2	FISHER	Cat#NB100-1277SS; Lot#S3-E190816
anti-Col1a1	Abcam	Cat#Ab21286; Lot#GR3186934-8
anti-Pou5f1	Abcam	Cat#Ab181557; Lot#GR219675-33
anti-tdTomato	Origene	Cat#AB8181-200

Id1	Proteintech	Cat#18475-1-AP
**Chemicals and Reagents**		
IHC Antigen Retrieval Solution	Invitrogen	Cat#00-4955-58; Lot#2085704
Trypsin-EDTA (0.25%)	Fisher	Cat#25-200-056
DAPI	Sigma-Aldrich	Cat#D9542
Diphtheria Toxin from Corynebacterium diphtheriae	Sigma-Aldrich	Cat#D0564-1MG
Vectashied, Mounting medium	Vector Labs Inc.	Cat#H-1000
Tamoxifen	Sigma-Aldrich	Cat#T5648-5G; Lot#WXBC1801V
Corn oil	Sigma-Aldrich	Cat#C8267-500ML; Lot#MKBW9504V
**IHC and In situ Kits**		
Mouse and Rabbit Specific HRP/DAB (ABC) Detection IHC kit	Abcam	Cat#Ab64264; Lot#GR3200245-11
RNASCOPE 2.5 HD DUPLEX REAGENT KIT	ACDBio	Cat#322430; Lot#2004306
**Oligonucleotides**		
Mouse-Wnt6	ACDBio	Cat#401111
Mouse-Lgr5	ACDBio	Cat#312171-C2
Mouse-Gng13	ACDBio	Cat#462531
Mouse-Fmr1	ACDBio	Cat#496391-C2

Primary and Secondary antibodies used in the immunofluorescence and immunohistochemistry; Chemicals used in DT or Tamoxifen injection; mRNA probes and kits used in the in situ hybridization.

Supplemental Datasets

Dataset S1. Mean mUMI (milliUMI)/cell for each germ cell cluster in Figure 2B

Gene name, cluster 0 (Pre-meiotic), cluster 1 (Pre-leptotene), cluster 2 (Leptotene), cluster 3 (Zygotene), cluster 4 (Pachytene), cluster 5 (Diplotene), cluster 6 (Dictyate), cluster 7 (Dying/nurse cell).

Dataset S2. Mean mUMI (milliUMI)/cell for each somatic cell cluster in Figure 3B

Gene name, cluster 0 (Epithelial_0), cluster 1 (Epithelial_1), cluster 2 (Epithelial_2), cluster 3 (Epithelial_3), cluster 4 (Bipotential), cluster 5 (B Pre-granulosa_0), cluster 6 (E Pre-granulosa_0), cluster 7 (B Pre-granulosa_1), cluster 8 (E Pre-granulosa_1), cluster 9 (B Pre-granulosa_2), cluster 10

(E Pre-granulosa_2), cluster 11 (B Pre-granulosa_3), cluster 12 (E Pre-granulosa_3), cluster 13 (B Pre-granulosa_4), cluster 14 (E Pre-granulosa_4), cluster 15 (B Granulosa_wave1), cluster 16 (E Granulosa_wave2).

Dataset S3. Marker genes for each germ cell cluster

Gene name, p_val, avg_logFC, pct.1, pct.2, p_val_adj.

Tabs at the bottom indicate: cluster 0 (Premeiotic), cluster 1 (Pre-Leptotene), cluster 2 (Leptotene), cluster 3 (Zygotene), cluster 4 (Pachytene), cluster 5 (Diplotene), cluster 6 (Dictyate), cluster 7 (Dying/nurse cell).

Dataset S4. Marker genes for each somatic cell cluster

Gene name, p_val, avg_logFC, pct.1, pct.2, p_val_adj.

Tabs at bottom indicate: cluster 0 (Epithelial_0), cluster 1 (Epithelial_1), cluster 2 (Epithelial_2), cluster 3 (Epithelial_3), cluster 4 (Bipotential), cluster 5 (B Pre-granulosa_0), cluster 6 (E Pre-granulosa_0), cluster 7 (B Pre-granulosa_1), cluster 8 (E Pre-granulosa_1), cluster 9 (B Pre-granulosa_2), cluster 10 (E Pre-granulosa_2), cluster 11 (B Pre-granulosa_3), cluster 12 (E Pre-granulosa_3), cluster 13 (B Pre-granulosa_4), cluster 14 (E Pre-granulosa_4), cluster 15 (B Granulosa_wave1), cluster 16 (E Granulosa_wave2).

Dataset S5. Genes significantly enriched in Epithelial or Bipotential Pre-Granulosa Cells.

Genes significantly enriched in either the EPG pathway (left, clusters 10, 12, 14, 16) or the BPG pathway (right, clusters 9, 11, 13, 15). Cluster names are listed below the Cluster numbers (Fig. 3B). For mean expression levels see Dataset S2.

Supplemental Figure Legends

Figure S1. Expression of marker genes in perinatal ovaries.

(A) Ovaries were stained for N2rf2, Foxl2, and the oocyte marker DDX4 at E12.5 and E18.5. (B) Ovaries were stained for Col1a1 and DDX4 at E14.5, and P2. DAPI (nuclear DNA). At E18.5, the Nr2f2-positive mesenchymal cells align in rows that have been likened to cords. Staining mesenchymal cells located between groups of cysts with Col1a1 further highlighted these ovarian subdomains, each of which contains several cysts or primordial follicles in E14.5 and P2 ovaries (Figure S1B). (C) Immunofluorescence of E12.5, E13.5, and E14.5 ovaries stained for Pou5f1 and Sycp3, showing the anterior-posterior expression gradient (yellow arrows). boxes: location of regions magnified at right. (D) Quantification of Pou5f1 and Sycp3 expression in fetal germ cells.

Figure S2. Heat map of differentially expressed genes for each germline cluster.

(A) Heat map depicting differentially expressed genes in each germ cell cluster in Fig. 2B (names at top) compared to all other clusters. Colored bars represent UMI expression levels of individual cells to provide information on observed variation in expression, including statistical noise. Genes names are indicated to the right of the heat map. Color scheme is based on z-score distribution from -2 (blue) to 2 (orange).

Figure S3. Heat map of differentially expressed genes for each somatic cell cluster.

(A) Heat map depicting differentially expressed genes in each somatic cell cluster in Fig. 3B compared to all other clusters. Colored bars represent UMI expression levels of individual cells to provide information on observed variation in expression, including statistical noise. Genes names are indicated to the right of the heat map. Color scheme is based on z-score distribution from -2 (blue) to 2 (orange).

Figure S4. Common and enriched BPG and EPG genes from E16.5-P5

(A) The Venn diagrams show the number of common genes in both BPG and EPG pathway, and the number of enriched genes in each pathway from E16.5 to P5. The box at the right lists some top expressed marker genes in each pathway. See Dataset S5 for specific genes in each class.

Figure S5. Lgr5+ pregranulosa cells from the surface epithelium no longer contribute to second wave follicles at P1.

(A) Schematic of the lineage tracing strategy. $Lgr5^{CreERT2/+}$ mice were crossed to $R26R^{tdT/tdT}$ reporter mice. Tamoxifen (Tmx) was administrated at P1, and samples were collected at P2 and P6. (B, C) Lineage tracing of Lgr5+ pregranulosa cell progeny showed that tdTomato+ cells remain at the ovary surface, and do not contribute significantly to second wave follicles by either P2 or P6. Compare to Figure 5F.

Fig. S1

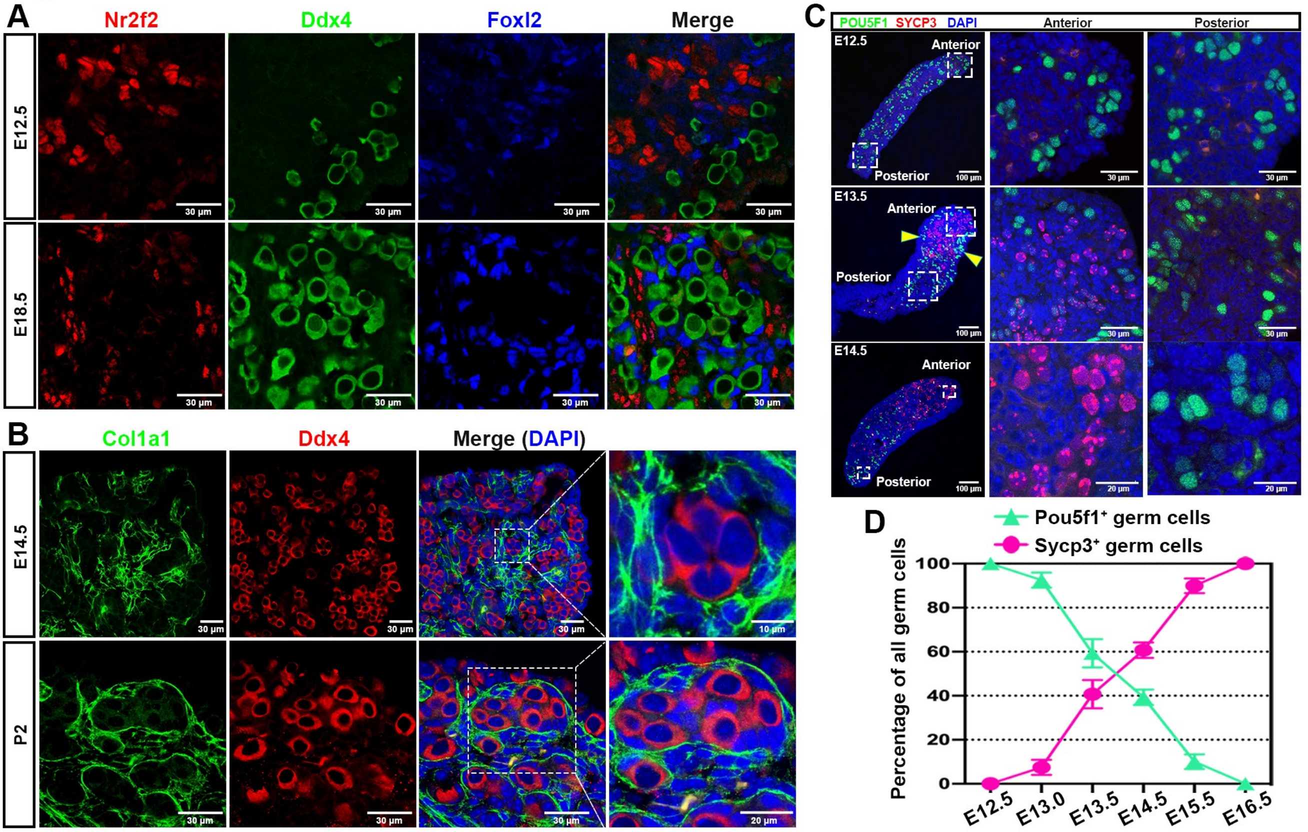
A
Nr2f2
Ddx4
Foxl2
Merge
E12.5
E18.5
30 μm
B
Col1a1
Ddx4
Merge (DAPI)
E14.5
P2
30 μm
10 μm
20 μm
C
POU5F1 SYCP3 DAPI
Anterior
Posterior
E12.5
E13.5
E14.5
100 μm
D
Pou5f1⁺ germ cells
Sycp3⁺ germ cells
Percentage of all germ cells
0
20
40
60
80
100
E12.5
E13.0
E13.5
E14.5
E15.5
E16.5

Fig. S2

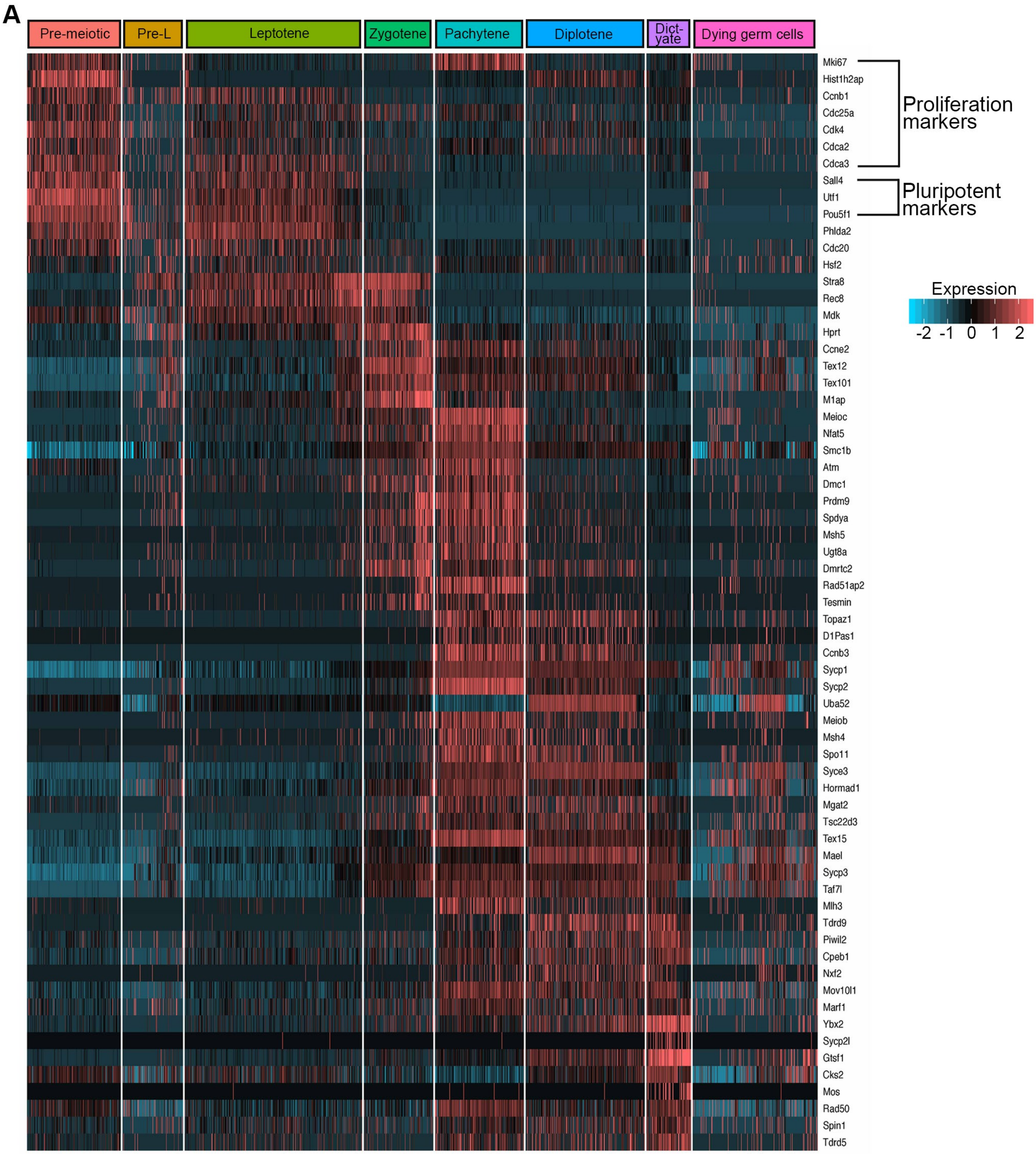

A
Pre-meiotic
Pre-L
Leptotene
Zygotene
Pachytene
Diplotene
Dict-yate
Dying germ cells
Mki67
Hist1h2ap
Ccnb1
Cdc25a
Cdk4
Cdca2
Cdca3
Sall4
Utf1
Pou5f1
Phlda2
Cdc20
Hsf2
Stra8
Rec8
Mdk
Hprt
Ccne2
Tex12
Tex101
M1ap
Meioc
Nfat5
Smc1b
Atm
Dmc1
Prdm9
Spdya
Msh5
Ugt8a
Dmrtc2
Rad51ap2
Tesmin
Topaz1
D1Pas1
Ccnb3
Sycp1
Sycp2
Uba52
Meiob
Msh4
Spo11
Syce3
Hormad1
Mgat2
Tsc22d3
Tex15
Mael
Sycp3
Taf7l
Mlh3
Tdrd9
Piwil2
Cpeb1
Nxf2
Mov10l1
Marf1
Ybx2
Sycp2l
Gtsf1
Cks2
Mos
Rad50
Spin1
Tdrd5
Proliferation markers
Pluripotent markers
Expression
-2 -1 0 1 2

Fig. S3

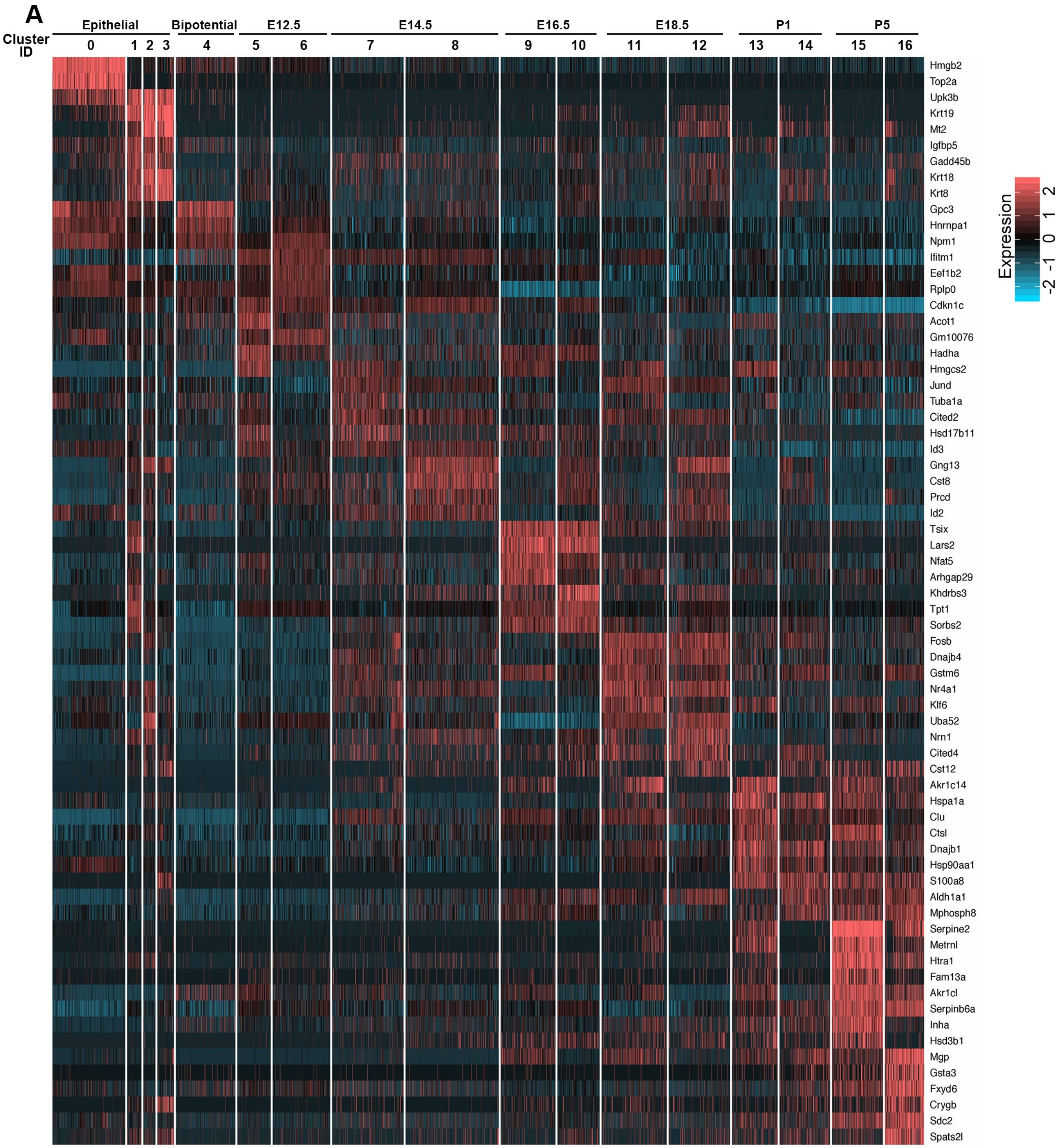
A
Epithelial
Bipotential
E12.5
E14.5
E16.5
E18.5
P1
P5
Cluster ID
0
1
2
3
4
5
6
7
8
9
10
11
12
13
14
15
16
Hmgb2
Top2a
Upk3b
Krt19
Mt2
Igfbp5
Gadd45b
Krt18
Krt8
Gpc3
Hnrnpa1
Npm1
Ifitm1
Eef1b2
Rplp0
Cdkn1c
Acot1
Gm10076
Hadha
Hmgcs2
Jund
Tuba1a
Cited2
Hsd17b11
Id3
Gng13
Cst8
Prcd
Id2
Tsix
Lars2
Nfat5
Arhgap29
Khdrbs3
Tpt1
Sorbs2
Fosb
Dnajb4
Gstm6
Nr4a1
Klf6
Uba52
Nrn1
Cited4
Cst12
Akr1c14
Hspa1a
Clu
Ctsl
Dnajb1
Hsp90aa1
S100a8
Aldh1a1
Mphosph8
Serpine2
Metrnl
Htra1
Fam13a
Akr1cl
Serpinb6a
Inha
Hsd3b1
Mgp
Gsta3
Fxyd6
Crygb
Sdc2
Spats2l
Expression
2
1
0
-1
-2

Fig. S4

A

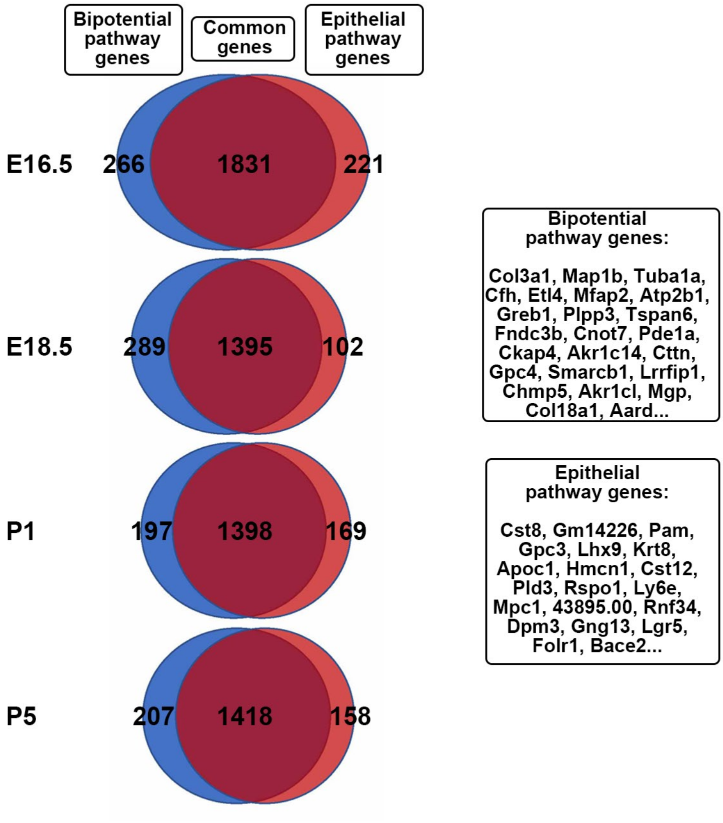
Bipotential pathway genes
Common genes
Epithelial pathway genes
E16.5
266
1831
221
E18.5
289
1395
102
P1
197
1398
169
P5
207
1418
158
Bipotential pathway genes:
Col3a1, Map1b, Tuba1a, Cfh, Etl4, Mfap2, Atp2b1, Greb1, Plpp3, Tspan6, Fndc3b, Cnot7, Pde1a, Ckap4, Akr1c14, Cttn, Gpc4, Smarcb1, Lrrfip1, Chmp5, Akr1cl, Mgp, Col18a1, Aard...
Epithelial pathway genes:
Cst8, Gm14226, Pam, Gpc3, Lhx9, Krt8, Apoc1, Hmcn1, Cst12, Pld3, Rspo1, Ly6e, Mpc1, 43895.00, Rnf34, Dpm3, Gng13, Lgr5, Folr1, Bace2...

Fig. S5

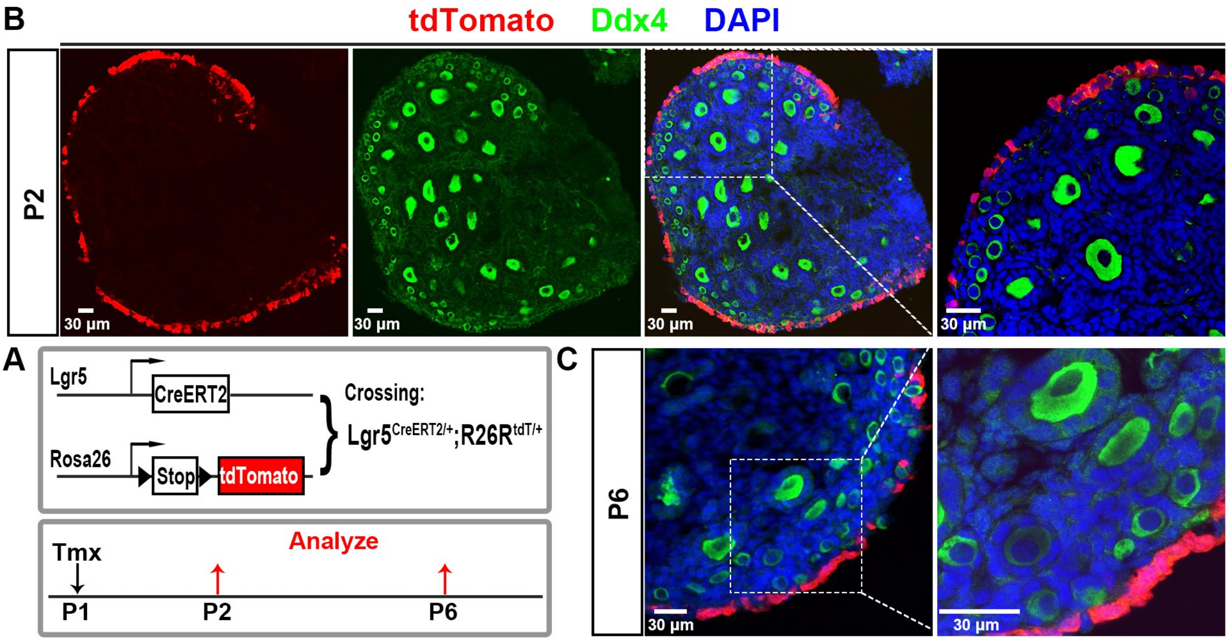
B
tdTomato Ddx4 DAPI
P2
30 µm
30 µm
30 µm
30 µm
A
Lgr5
CreERT2
Rosa26
Stop
tdTomato
Crossing:
Lgr5CreERT2/+;R26RtdT/+
Tmx
Analyze
P1
P2
P6
C
P6
30 µm
30 µm
